# Generation and characterization of feline arterial and venous endothelial cell lines for the study of the vascular endothelium

**DOI:** 10.1186/1746-6148-9-170

**Published:** 2013-08-29

**Authors:** Dominique AJ Olyslaegers, Lowiese MB Desmarets, Annelike Dedeurwaerder, Hannah L Dewerchin, Hans J Nauwynck

**Affiliations:** 1 Department of Virology, Parasitology and Immunology, Faculty of Veterinary Medicine, Ghent University, Salisburylaan 133, B-9820 Merelbeke, Belgium

**Keywords:** Endothelial cells, Feline, Immortalization, SV40LT, hTERT, Aorta, Vena cava

## Abstract

**Background:**

The *in vitro* culture of endothelial cells (ECs) is an indispensable tool for studying the role of the endothelium in physical and pathological conditions. Primary ECs, however, have a restricted proliferative lifespan which hampers their use in long-term studies. The need for standardized experimental conditions to obtain relevant and reproducible results has increased the demand for well-characterized, continuous EC lines that retain the phenotypic and functional characteristics of their non-transformed counterparts.

**Results:**

Primary feline ECs from aorta and vena cava were successfully immortalized through the successive introduction of simian virus 40 large T (SV40LT) antigen and the catalytic subunit of human telomerase (hTERT). In contrast to the parental ECs, the transformed cells were able to proliferate continuously in culture. Established cell lines exhibited several inherent endothelial properties, including typical cobblestone morphology, binding of endothelial cell-specific lectins and internalization of acetylated low-density lipoprotein. In addition, the immortalization did not affect the functional phenotype as demonstrated by their capacity to rapidly form cord-like structures on matrigel and to express cell adhesion molecules following cytokine stimulation.

**Conclusion:**

The ability to immortalize feline ECs, and the fact that these cells maintain the EC phenotype will enable a greater understanding of fundamental mechanisms of EC biology and endothelial-related diseases. Furthermore, the use of cell lines is an effective implementation of the 3-R principles formulated by Russel and Burch.

## Background

Endothelial cells line the inner surface of blood vessels. In this strategic position, they play a key role in a large number of important physiological processes, such as regulation of vascular tone and blood flow, fluid and solute exchange, haemostasis and coagulation, inflammatory responses and angiogenesis. Furthermore, the endothelium is actively involved in a wide variety of pathological processes, including tumor invasion, atherosclerosis, arthritis, thrombosis, and vasculitis [1,2]. In order to investigate the role of ECs in these events, it has been proven valuable to optimize techniques to isolate, culture and characterize these cells *in vitro*. Substantial data supports a central role of endothelial dysfunction and/or injury in the pathogenesis of many diseases in cats, including feline infectious peritonitis (FIP), feline immunodeficiency virus (FIV) and several neoplasms [3-5]. In addition, numerous interest has arisen in feline endothelial cell cultures as they provide representative *in vitro* systems for the study of several vascular disorders in human, such as myocardial ischemia and reperfusion injury [6]. On top, FIV infection in cats represents a well-established animal model in the study of human immunodeficiency virus (HIV)-1 infection. The availability of feline endothelial cell cultures would therefore offer an important asset to gain further understanding in endothelial-related diseases in cats and to address questions related to vascular pathophysiology in humans through the use of feline models [7].

In the past, ECs were considered to be a homogeneous cell population that functions merely as a passive physical barrier between blood and tissue. The last decade, it became increasingly apparent that ECs display significant heterogeneity in phenotype, function, antigenic composition and biological behavior depending on the vascular system they originate from. Differences not only exist between macro- and microvascular ECs, but arterial and venous ECs also differ intrinsically [8]. Therefore, when studying the biological characteristics of a disease, it is desirable to use ECs isolated from vessels of the appropriate size and functionality, that originate from the proper anatomical compartment.

Freshly isolated cells are well differentiated and provide cultures that have characteristics very close to the tissue of origin. Therefore, much of the knowledge on properties and functions of the vascular endothelium has been obtained from primary EC cultures. However, the use of primary ECs presents various disadvantages. They frequently require special culture substrata, growth factors, cofactors and high serum concentrations. Furthermore, like most somatic cells, ECs undergo only a predetermined and finite number of cell divisions in culture. Thereafter, cells enter an irreversible proliferation arrested state, referred to as replicative senescence and finally die [9]. Because of this limited lifespan of primary cells, researchers frequently need to re-establish fresh cultures. The isolation of ECs often implies labor intensive procedures with varying success and reproducibility. Moreover, the behavior of cells may differ considerably from batch to batch due to their multidonor origin, what makes the comparison of experimental results obtained with different EC isolates questionable. The above limitations of primary cells and the need for consistent material throughout long-term studies, have increased the demand for continuously growing (immortalized), well-characterized EC lines stably presenting endothelial properties. Efforts to extend the *in vitro* lifespan of cells have frequently focused on their transformation with viral oncogenes from DNA tumor viruses. The common mode of action of these viral oncoproteins is to bind and inactivate the protein products of the cell cycle regulatory genes p53 and retinoblastoma (Rb), allowing cells to overcome senescence signals and to continue proliferation [10,11]. Of this type, overexpression of the gene for Simian Virus 40 large T antigen has been the most widely applied technique to bypass replicative senescence. Nonetheless, in most reports the additional lifespan of the post-senescent cell lines expressing SV40LT is still restricted by another barrier called crisis. The onset of crisis typically coincides with critically short telomeres accompanied by chromosomal instability and widespread apoptosis [12]. Therefore, ectopic expression of the human telomerase reverse transcriptase (hTERT) gene, being the catalytic component of telomerase, has been proposed as an alternative method to hurdle replicative senescence. Cells with reconstituted telomerase activity maintain sufficient telomere lengths that allows cells to not only circumvent replicative senescence, but escape from crisis and become truly immortal [13]. Nevertheless, immortalization of cells by induction of telomerase activity alone is still controversial and several cell types even seem refractory to the hTERT immortalization protocol [14]. Meanwhile, coexpression of hTERT and a viral oncogene, like SV40LT antigen, has proven to be effective and efficient to immortalize a number of cell types including endothelial cells [15,16]. Besides, cell lines transformed with an oncogene/hTERT combination have significantly more genomic stability than cell lines immortalized with an oncogene alone [15]. A diverse range of gene delivery systems have been developed to insert genetic material into cells. Since efficient non-viral transfection of primary cells is still a challenge, gene transfer into primary cells is restricted to the use of viral vectors. A variety of recombinant viral vectors have been tailored to their specific applications. Recently, the lentiviral vector system has gained popularity as it can actively pass through the nuclei membrane enabling the transduction of not only dividing but also non-dividing cells. In addition, lentiviral vectors will integrate into the host cell genome, giving rise to permanent and stable gene expression [17]. These properties make this vector the most suitable vehicle for transducing the feline EC cultures with the immortalization genes.

Present work details the successful isolation and culture of ECs from feline aorta and vena cava, using relatively straightforward techniques. Next, considering above mentioned immortalization paradigms, immortalized EC lines were established by transducing these primary EC cultures with a combination of SV40LT antigen and hTERT using lentiviral-based vectors. Finally, cell lines were extensively characterized and compared with their non-transformed counterparts for the expression of critical phenotypic EC markers. In addition, the response of the immortalized EC lines to known inflammatory stimuli and their capacity to form tube-like capillary structures on matrigel was assessed to demonstrate a differentiated functional state similar to their parental cells. Well-characterized feline endothelial cell lines with a known and constant functional profile can facilitate studies to investigate the role of ECs in vascular physiology and pathology in cats and humans.

## Methods

### Primary EC cultures

Cats are euthanized in practice for variable reasons. The use of tissues from these cats was approved by the ethical committee of the Faculty of Veterinary Medicine, Ghent University (application EC2012/043). In collaboration with practice veterinarians, vessels for EC isolation were collected from freshly euthanized animals, after informal consent of the cats’ owners. All isolation procedures were performed in a laminar flow hood using sterile techniques and supplies.

#### Aorta

Endothelial cells were directly isolated after euthanasia of the cat with an overdose of 20% SodiumPentobarbital (1 ml/1.5 kg; Kela Laboratories, Hoogstraten, Belgium). After opening the chest and the abdomen, a blunt needle was inserted in the aorta ascendens and immobilized with a hemostatic clamp. A small incision was made in the aorta wall just before it branches into the iliac arteries. After rinsing with phosphate buffered saline (PBS; Vel chemicals, UCB, Brussels, Belgium) to remove any residual blood, a blunt needle was placed in the latter incision and secured by a hemostatic clamp. Important branches of the aorta were pinched off. Prewarmed enzyme mixture of 0.1% type I collagenase (Invitrogen, Paisley, UK) and 0.12% dispase (Sigma-Aldrich, St. Louis, MO, USA) in Dulbecco’s Modified Eagle’s Medium (DMEM; Gibco BRL, Merelbeke, Belgium), was infused into the vessel. During enzyme incubation, the vessel was gently ‘massaged’ to facilitate EC detachment. After 10 min of incubation, the enzyme solution, containing the ECs, was flushed from the aorta by perfusion with DMEM (37°C) from one syringe through the vessel into the other syringe. The incubation and perfusion steps were repeated four times with introduction of fresh enzyme solution each time. The effluent was collected in chilled centrifuge tubes containing fetal calf serum (FCS; Gibco BRL). The cells were sedimented at 200 × g at 4°C for 10 min. The supernatant was discarded and the pellet washed 2 times with cold DMEM. The final pellet was resuspended in endothelial growth medium and plated onto 0.5% gelatin-coated culture dishes (Nunc A/S, Roskilde, Denmark). Endothelial growth medium consisted of DMEM supplemented with 10% FCS, 100 U/ml penicillin, 0.1 mg/ml streptomycin, 0.1 mg/ml gentamycin, 1 mM sodium pyrovate, 1% non-essential amino acids 100× (Gibco BRL), 50 μg/ml endothelial cell growth supplement (ECGS; Biomedical Technologies Inc., Stoughton, MA, USA) and 10 U/ml heparin (Leo, Zaventem, Belgium). Cultures were maintained at 37°C in a humidified incubator with 5% CO_2_. The medium was changed within 24 h of initial plating, thereafter at 48–72 h intervals.

#### Vena cava

Immediately after euthanasia the vena cava inferior was aseptically removed from the cats’ abdominal cavity and transferred to a petri dish. Periadventitial fat and connective tissue was carefully stripped by blunt dissection. One end of the vein was cannulated with a blunt needle, secured by a hemostatic clamp. After rinsing with PBS the other end of the vessel was clamped, using a hemostat. The segment was filled with prewarmed collagenase/dispase (0.1%/0.12%) solution until there was moderate distention of the vessel. After an incubation time of 20 min, the occluding hemostatic clamp was opened. A fresh cut was made to remove the crushed part of the vessel. The endothelial cells, liberated by the enzymes, were obtained by flushing the vessel with DMEM (37°C). The effluent was collected into sterile syringes and transferred into chilled centrifuge tubes with FCS. Cells were pelleted by centrifugation at 200 × g at 4°C for 10 min. After 2 additional washes with cold DMEM, the pellet was resuspended in endothelial growth medium, plated on 0.5% gelatin-coated plastic ware and incubated at 37°C in a 5% CO_2_-air atmosphere. After overnight incubation, cell debris and non-adherent cells were washed away with prewarmed DMEM and fresh medium was added. Thereafter, cells were refed every 2–3 days.

### Immortalization of endothelial cells

Cell lines were created by sequentially introducing the simian virus 40 large T antigen followed by human telomerase reverse transcriptase into primary EC cultures. Midconfluent, proliferating primary cultures were first exposed to the recombinant lentiviral vector containing the sequence encoding the SV40LT transforming protein (Applied Biological Materials Inc., Richmond, BC, Canada) in the presence of polybrene (8 μg/ml, Applied Biological Materials Inc.). To avoid cytotoxicity, the viral supernatant was diluted after 30 min with heparin free EC growth medium (1:1) and further incubated overnight. The medium was replaced by fresh complete growth medium the next day and then every second day until cells reached confluence. After 3 passages, the polyclonal populations of SV40LT expressing cells, overcoming senescence, were infected with recombinant lentiviral vector carrying the hTERT gene (Applied Biological Materials Inc.), as described above. This vector also contains the puromycin resistance gene as a selection marker. At 72 h post-transduction, cells were incubated in their regular growth medium containing puromycin (10 μg/ml; Applied Biological Materials Inc.) to select for stable hTERT transduced cells. Selection was carried out until the cultures were devoid of nonresistant cells (< 14 days) and surviving cells were further expanded in standard medium and routinely passaged at a 1:3 split ratio using 10% trypsin (Sigma-Aldrich)/1% versene (Vel chemicals) in PBS.

### Characterization of primary endothelial cells and EC lines

Endothelial cells isolated from aorta and vena cava and the immortalized ECs established from these primary cultures were characterized in a number of ways. Cell characterization studies of the immortalized ECs were carried out with early-passage cells (P6-P10) and repeated with cells at a higher passage number (P28-P31) to ensure the stability of the endothelial traits.

#### Morphology

Cultures were examined daily by inverted light microscopy and photographed in phase-contrast (IX50; Olympus, Tokyo, Japan).

#### Cell characterization by immunofluorescence

##### von Willebrand’s factor (vWF) immunofluorescence

The presence of vWF was determined by direct immunofluorescence using fluorescein isothiocyanate (FITC)-labeled sheep polyclonal anti- human vWF antibodies (AbD Serotec, Raleigh, NC). ECs were seeded on glass coverslips and allowed to attach and grow overnight. After removal of the medium, cells were fixed with 4% paraformaldehyde (PF) for 10 min at room temperature (RT). Following 2 washes in PBS, the cells were permeabilized with 0.1% Triton X-100 (Sigma-Aldrich) for 2 min at RT. After being rinsed twice in PBS, cells were incubated with a 1:50 dilution of the polyclonal antibody at 37°C for 1 h. Cells were washed twice with PBS, and the nuclei were counterstained with Hoechst 33342 (Molecular Probes, Eugene, Oregon, USA) for 10 min at 37°C. After an additional wash in PBS the coverslips were inverted over a drop of glycerin/PBS (0.9 : 0.1, v/v) with 2.5% 1,4-diazabicyclo(2,2,2) octane (Janssen Chimica, Beerse, Belgium) and analysed using confocal microscopy (Leica Microsystems DMRBE, Wetzlar, Germany).

##### *Ulex europaeus* agglutinin-1 (UEA-1) expression

The presence of UEA-1 binding antigen was evaluated on endothelial cells grown on coverslips. PF (4%)-fixed cells were incubated with biotin-labeled UEA-1 lectin (Sigma-Aldrich) at a concentration of 500 μg/ml for 1 h at 37°C. After 2 rinses with PBS the lectin was revealed by subsequent incubation with streptavidin-Texas Red (1:100; Molecular Probes) for 1 h at 37°C. Cells were washed with PBS, and the cell nuclei were stained with Hoechst for 10 min at 37°C. After final rinses with PBS, coverslips were mounted in glycerin/PBS (0.9:0.1, v/v) with 2.5% 1,4-diazabicyclo (2,2,2) octane and examined for fluorescence with a confocal microscope. To exclude false positives produced by nonspecific binding of the secondary antibody, controls lacking the lectin were included.

##### Uptake of acetylated low-density lipoprotein (Ac-LDL)

The presence of scavenger receptors for acetylated low density lipoprotein on EC was detected using 1,1-diocatadecyl-3,3,3′,3′-tetramethyl-indocarbocyanine perchlorate Ac-LDL (DiI-Ac-LDL; Biomedical Technologies Inc.). Near confluent EC cultures grown on coverslips were incubated with 10 μg/ml DiI-Ac-LDL in complete growth medium for 4 h in a humified 5% CO_2_/air incubator at 37°C. After the incubation period, cells were rinsed 3 times with probe free medium and fixed with paraformaldehyde (4%) for 10 min at RT. Nuclei were stained with Hoechst 33342 (10 min, RT). Coverslips were mounted in glycerin/PBS (0.9:0.1, v/v) with 2.5% 1,4-diazabicyclo(2,2,2) octane and DiI-Ac-LDL uptake was visualized with a confocal microscope.

##### Expression of cytoskeletal proteins

To assess the expression of the different cytoskeletal proteins, immunostainings were performed against vimentin, desmin, and alpha smooth muscle actin (SMA). Cells were fixed with 4% PF in PBS for 10 min at RT followed by permeabilization with 0, 1% Triton X-100 for 2 min at RT. Cells were incubated with the appropriate primary antibody dilutions containing 10% normal goat serum (NGS) for 1 h at 37°C. Primary monoclonal antibodies were anti-vimentin (1:100; Lab Vision Corporation, Fremont, CA, USA), anti-desmin (1:100; Dako, Glostrup, Denmark) and anti-SMA (1:100; Dako). After 2 washes in PBS, Texas Red-conjugated goat anti-mouse IgG (1:100; Molecular Probes) was added for 1 h at 37°C to detect the binding of the primary antibodies. Nuclei were stained with Hoechst 33342 for 10 min at RT. The coverslips were washed 2 times in PBS, mounted and examined for fluorescence. Negative controls, where the primary antibodies were substituted with isotype matched irrelevant monoclonal antibodies at equivalent concentration, were routinely performed.

##### Immunodetection of SV40LT antigen and hTERT in EC lines

Immortalized ECs were analyzed for the co-expression of hTERT and SV40L T antigen by indirect immunofluorescence. Cells grown on glass coverslips were fixed with 4% PF and permeabilized with 0.1% Triton X-100. Cells were first incubated with polyclonal rabbit antibodies against hTERT (Applied Biological Materials Inc.) containing 10% NGS for 1 h at 37°C. Binding of the primary antibodies was revealed by subsequent incubation with FITC-conjugated goat anti-rabbit IgG antibodies (Molecular Probes) for 1 h at 37°C. Next, cells were incubated with monoclonal antibodies specific for SV40LT antigen (Applied Biological Materials Inc.) containing 10% NGS, followed by goat anti-mouse-Alexa fluor 594-labeled antibodies (Molecular Probes), each for 1 h at 37°C. Nuclei were stained and coverslips mounted as described above.

#### Capillary-like tube formation assay on matrigel

Matrigel (BD biosciences, Bedford, MA, USA) was allowed to thaw on ice overnight. Prechilled 24-well culture dishes were coated on ice with 300 μl matrigel per well. Gels were allowed to solidify for 30 min at 37°C. Endothelial cells were trypsinized and 10^5^ cells, resuspended in 500 μl endothelial growth medium, were plated onto these gels. The plates were returned to the incubator and incubated for 24 h. Morphological changes were periodically monitored and photographed using an inverted phase-contrast microscope.

#### Quantification of cell adhesion molecule expression in response to tumor necrosis factor-alpha (TNF-α)

An enzyme-linked immunosorbent assay (ELISA) was used to quantify changes in surface expression of the endothelial cell adhesion molecules E-selectin, intercellular adhesion molecule-1 (ICAM-1), and vascular cell adhesion molecule-1 (VCAM-1) in response to TNF-α. Homogenous EC monolayers, and therefore single-cell suspensions as starting material, are necessary to obtain accurate results with this test. Since the isolation of primary ECs resulted in cell clusters, which was necessary for sufficient outgrowth of the isolated cells, and subpassage of the primary cultures was difficult (aortic ECs) or even impossible (venous ECs), the ELISA was only performed on the immortalized ECs. Immortalized ECs were seeded in normal growth medium on gelatin-coated 96-well plates (BD biosciences) at a density of 5000 cells/well. At 3 days post confluence, cultures were incubated for 6 h (E-selectin) or 12 h (ICAM-1 and VCAM-1) with fresh growth medium supplemented with increasing concentrations of recombinant feline TNF-α (0.01, 0.1, 1, and 10 ng/ml; R&D Systems, Minneapolis, MN, USA) or vehicle control (PBS plus 0.5% bovine serum albumin; Sigma-Aldrich). After stimulation, cells were gently washed once with DMEM and incubated with monoclonal antibodies to ICAM-1 (1:200; santa cruz biotechnology, Santa Cruz, CA, USA), E-selectin and VCAM-1 (1:200; Abd serotec) for 1.5 h on ice. After washing 3 times with DMEM, the EC monolayers were fixed with 1% PF for 5 min at RT. After three additional washes with PBS, cells were exposed to the secondary antibody, horseradish peroxidase-conjugated goat anti-mouse IgG (1:2000; Molecular Probes) for 1 h at RT. Finally, cells were triple washed with PBS containing 0.05% Tween 20 (Sigma-Aldrich) and binding of antibody was detected by the addition of 50 μl tetramethylbenzidine substrate solution (R&D systems). After exactly 15 min, the color reaction was stopped by the addition of 50 μl of 1 M H_2_SO_4_ and the optic density (OD) was measured at 450 nm using an ELISA reader (Thermo LabSystems, Beverly, MA, USA). Cells stained with isotype matched irrelevant monoclonal antibodies were used as background wells. All data points were performed in triplicate and results were expressed as mean with standard deviation (SD).

### Statistic analysis

The significance of differences in surface expression of cell adhesion molecules between vehicle control and TNF-α treatment at graded doses was calculated with the Mann–Whitney *U*-test (two-tailed). P values equal or lower than 0.05 were considered to be statistically significant. All statistical analyses were performed using SPSS 19.0 (SPSS Inc., Chicago, IL, USA).

## Results

### Cell morphology and growth characteristics

#### Aorta

**Figure 1:**
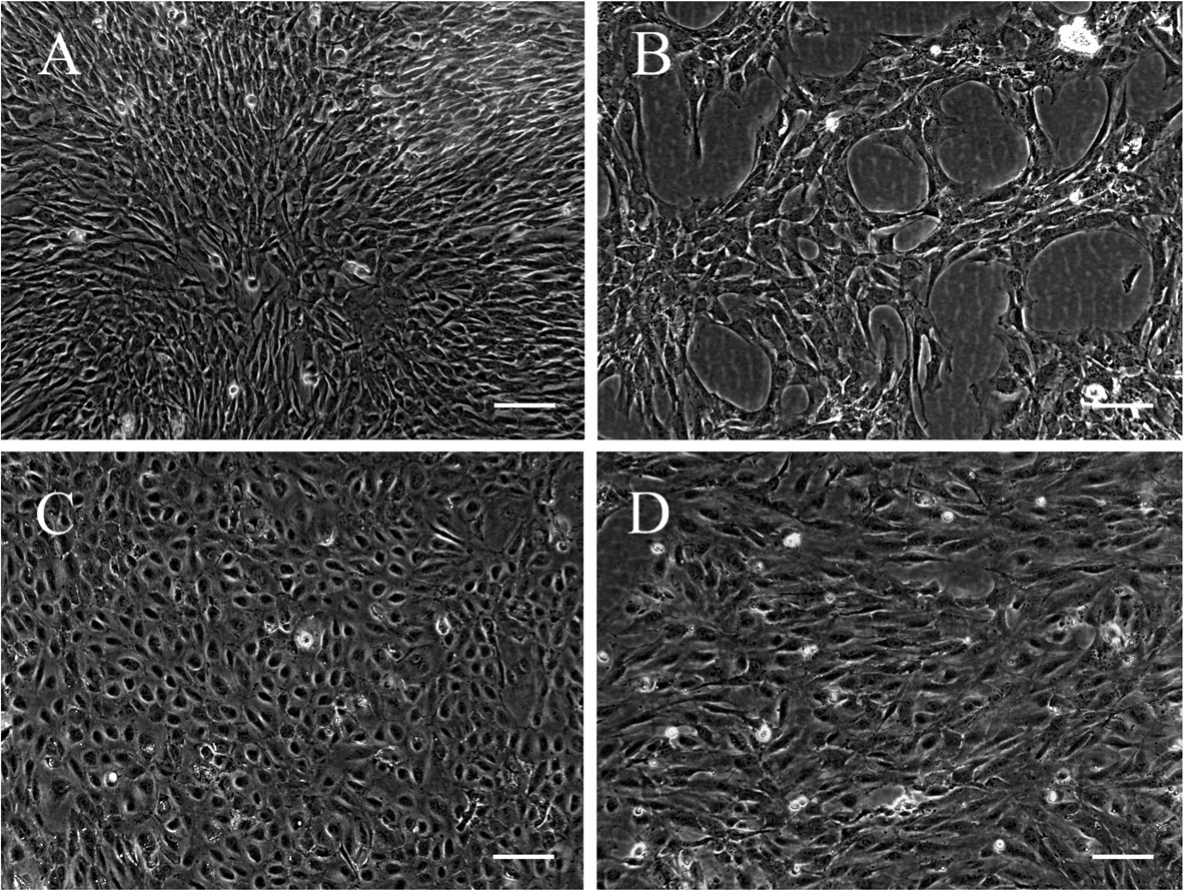
**Morphologic features of primary ECs and EC lines in culture demonstrated by phase contrast microscopy.** At confluence, primary aortic ECs show an elongated phenotype with no strict contact inhibition **(A)**. Immortalized aortic ECs form networks in sparse culture **(B)**. Confluent monolayers of primary venous ECs **(C)** and immortalized venous ECs **(D)** demonstrate a characteristic cobblestone-like appearance. Scale bar, 100 μm.

At the completion of the primary isolation, aggregates of 20–50 aortic ECs could be seen floating in the medium. Cells attached to tissue culture plates within 24 h. After another day, marked proliferation began as daughter cells dispersed from one another, indicating their amoeboid activity. At confluence, a tightly packed monolayer of slightly elongated cells with some overlapping of adjacent cells was evident (Figure 1A). Cells could be propagated for one, sometimes two, passages before reaching a state of replicative senescence.

Following the transduction of the primary ECs with the SV40LT antigen, there was a 2-week period of dramatically decreasing cell viability, followed by the emergence of viable colonies. The frequency of clone formation approximate 5×10^-5^ and these clones of fast growing cells were further expanded before hTERT transduction. Transformed aortic ECs divided faster and were able to grow in medium with a low concentration of FCS (0.5%) and without growth factors (heparin and ECGS) that does not support growth of non-transformed cells. Their morphology was essentially indistinguishable from that of primary endothelial cells. However, in sparse cultures they formed networks, as demonstrated in Figure 1B, and when allowed to become hyperconfluent, cells were capable of growing to a higher density and appeared smaller under these conditions. To date, transformed aortic cells has been passaged over 64 times over the span of 10 months without any change in morphology or growth characteristics.

#### Vena cava

Liberated cell clusters from the vena cava, each containing 3 to 7 ECs, adhered within 24 h. After one day, cells grew outward from the colonies by migration and proliferation. During this stage cells assumed an elongated morphology but formed a contact-inhibited cobblestone-like monolayer of polygonal cells at confluence (Figure 1C). By the first subculture, cells already demonstrated apparent senescene, characterized by slow growth, cellular enlargement and multinucleation.

Three attempts of transduction of independently derived primary venous ECs were necessary to generate SV40LT positive clones and the frequency of emergence of the transformed clones was in the order of 1-3×10^-6^. In contrast to the parental cells, a substrate of gelatin and the presence of high serum concentration and growth factors were not essential for the cell attachment and growth of the SV40LT/hTERT expressing cells. The cell line formed a monolayer of density-inhibited cells with a cobblestone-like morphology, comparable to their primary counterparts (Figure 1D). Cells have been maintained in culture for more than 11 months (53 passages) without any signs of senescence and with preserved characteristics.

### Cell characterization by immunofluorescence

#### von Willebrand Factor

**Figure 2:**
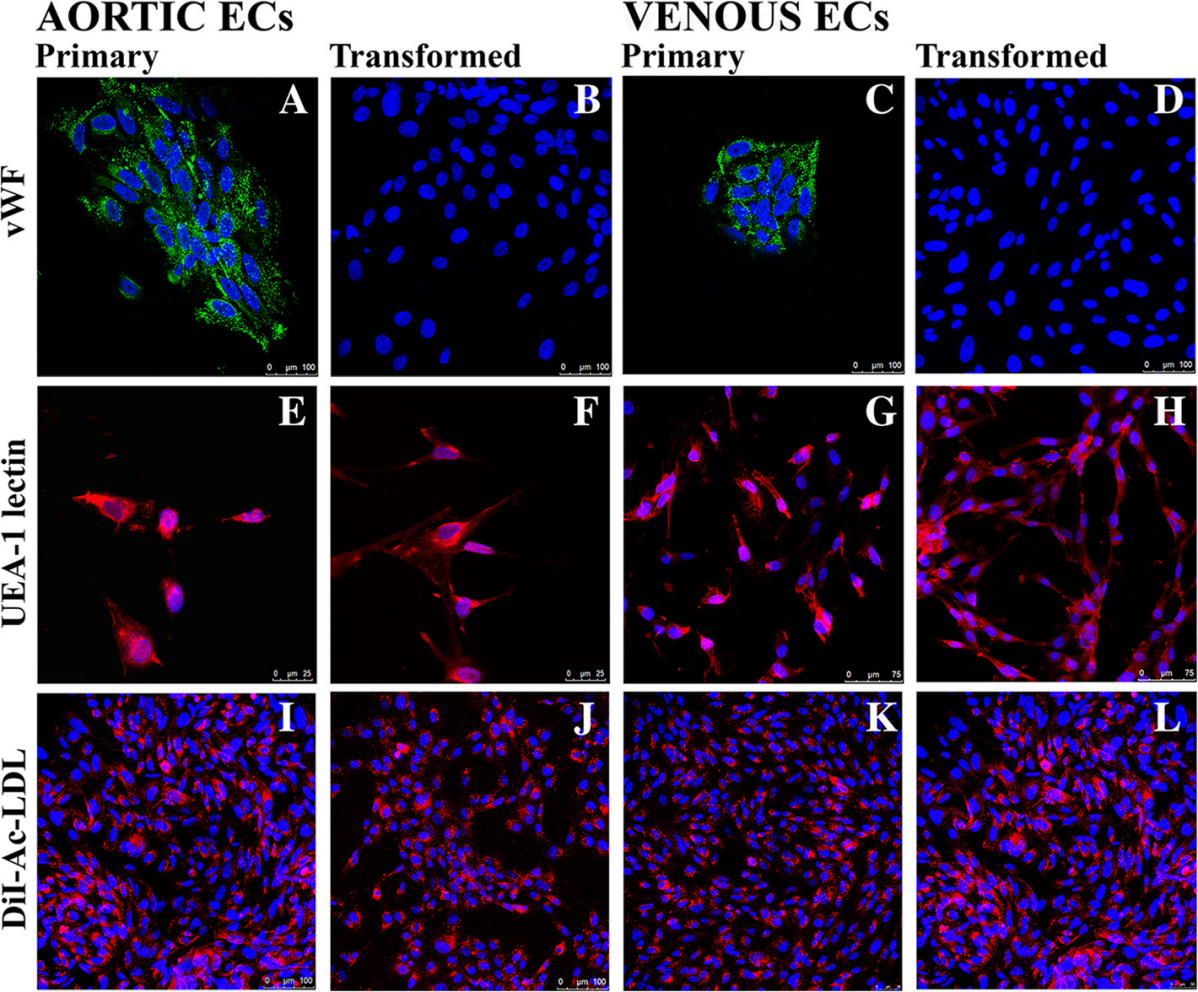
**Characterization of cultured primary ECs and EC lines.** Representative examples of immunofluorescence stainings of endothelial cell specific markers. Primary ECs **(A and C)** demonstrate an intense perinuclear granular immunofluorescence for vWF, whereas the transformed counterparts **(B and D)** completely lack vWF expression. Both non-transformed and transformed ECs **(E-H)** react with UEA-1 lectins, as a membrane-associated staining pattern was evident. Primary **(I and K)** and immortalized **(J and L)** ECs incorporate DiI-Ac-LDL to the same extent, resulting in an intense punctuate fluorescence with predominantly a perinuclear distribution.

Cells from primary EC cultures were strongly labeled with antiserum against the von Willebrand factor. At high magnification, small fluorescently labeled vesicles in a distribution consistent with that of Weibel-Palade (WP) bodies were evident (Figure 2A, C). Subcultures of non-transformed ECs were only weakly positive for vWF and did not contain WP bodies (data not shown). The production of vWF was completely extinguished in transformed cells (Figure 2B, D).

#### *Ulex europaeus* agglutinin-1

Endothelial cells were stained with lectin, a plant agglutinin derived from *Ulex europaeus* that recognize α-L-fucose-containing glycoproteins present on the surface of ECs. A cell membrane-associated staining pattern was clearly evident in primary ECs derived from aorta and vena cava (Figure 2E, G). A similar positive staining was observed in the transformed counterparts (Figure 2F, H).

#### Uptake of acetylated low-density lipoprotein

Primary ECs and EC lines were evaluated for their ability to metabolize Ac-LDL, a function mediated by specific receptors found on ECs and macrophages. Following 4 h incubation with DiI-Ac-LDL-supplemented media, a marked accumulation of the fluorescent lipoprotein was observed in all cultures of non-transformed (Figure 2I, K) and transformed cells (Figure 2J, L). The dot-like fluorescence appeared throughout the cell cytoplasm but was especially concentrated in the perinuclear region of the cells.

#### Expression of cytoskeletal proteins

To assess the intermediate filament protein profile, vimentin and desmin expression was detected by immunocytochemistry. The expression of the myofilament, smooth muscle alpha actin, was also examined. Vimentin was expressed abundantly and homogenously in the primary and SV40LT/hTERT expressing EC cultures, indicating their mesenchymal origin. Cultures lacked desmin and SMA expression, thereby excluding the possibility of smooth muscle cell and/or pericyte contamination.

#### Expression of SV40LT antigen and hTERT in EC lines

**Figure 3:**
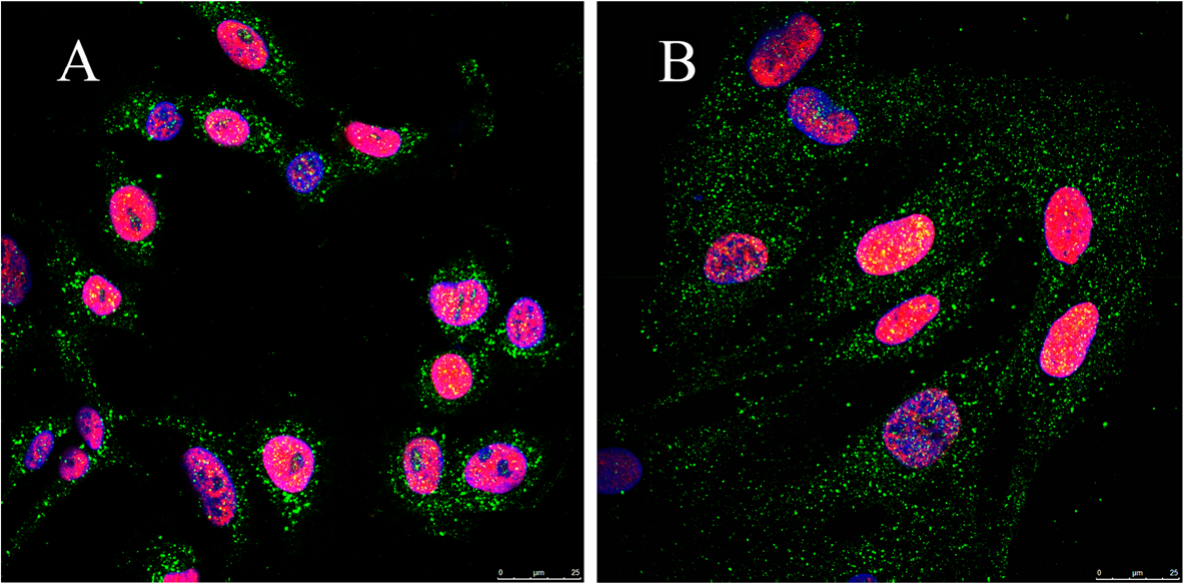
**Immunodetection of SV40LT antigen and hTERT in EC lines.** Aortic EC line **(A)** and venous EC line **(B)** were uniformly positive to nuclear SV40LT antigen (red) and cytoplasmic hTERT (green).

The success of transformation of primary EC cultures to continuous EC lines was confirmed by immunocytochemical staining against the two transduced immortalization genes. EC lines were uniformly positive to nuclear SV40LT antigen and cytoplasmic hTERT (Figure 3).

#### Capillary-like tube formation on matrigel

**Figure 4:**
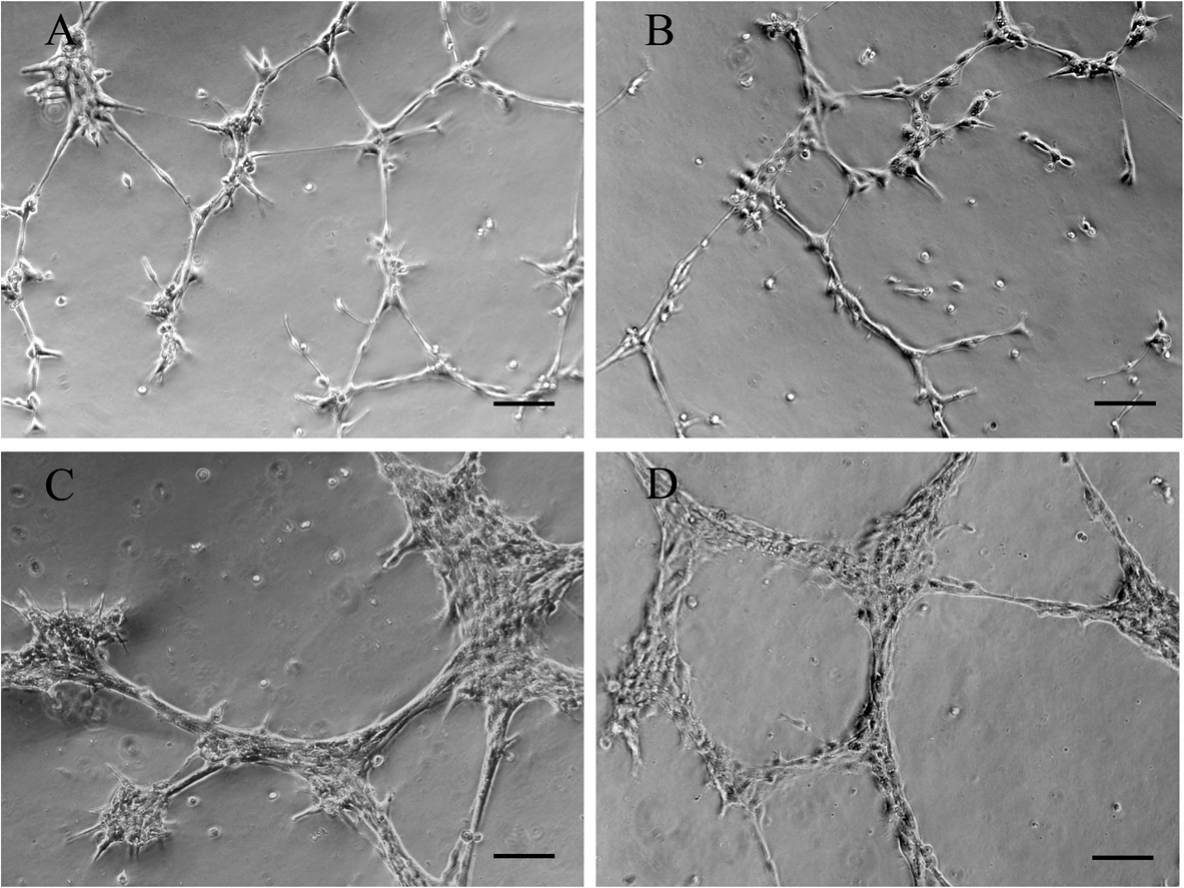
**Capillary-like tube formation on matrigel of primary ECs and EC lines.** Phase-contrast images showing angiogenic response of primary **(A)** and immortalized **(C)** aortic ECs and primary **(B)** and immortalized **(D)** venous ECs, 8 h after plating cells on 24-well plated precoated with matrigel at a density of 10^5^ cells/well. Primary ECs **(A-B)** mainly exhibited single-cell processes, whereas immortalized ECs **(C-D)** predominantly reorganized into thicker, multicellular cords. Scale bar, 100 μm.

Endothelial cells cultured on an appropriate extracellular matrix, such as matrigel, are capable of orienting into capillary-like structures, reminiscent of blood vessel angiogenesis *in vivo*. To evaluate whether transformed ECs retained the capacity to exhibit this endothelial function, cells were plated onto matrigel and the extent of tubulogenesis was assessed by light microscopic examination at different times after plating. Both EC lines were able to demonstrate an angiogenic response. Cells attached rapidly and immediately began to elongate and to align themselves into cellular arrays. The onset of tubule-like structure formation could be visualized as early as 1 h after plating, with more complex formation and extensive branching over the next hours. After 3 to 4 h of initial seeding the immortalized ECs had formed an interconnected network of anastomosing cells that by low power light microscopy had a honeycomb appearance. This tubule network only remained stable for 10 to 12 h after which it disintegrated. Although tube formation of immortalized ECs paralleled that of non-immortalized ECs, there were notable differences in both the time course and the composition of tube formation. Tubulogenesis of primary ECs was less rapid with elongated processes only observed 3 to 4 h after initial seeding and tube-like structure formation apparent after 6 to 8 h. Yet, the netlike structures in the non-transformed EC cultures maintained for more than 18 h. Tubules eventually retracted and at 24 h cells had clumped into a mass. Primary ECs also mainly exhibited single-cell processes (Figure 4A, B), whereas immortalized ECs predominantly reorganized into thicker and multicellular cords (Figure 4C, D).

#### Surface expression of cell adhesion molecules in response to TNF-α

**Figure 5:**
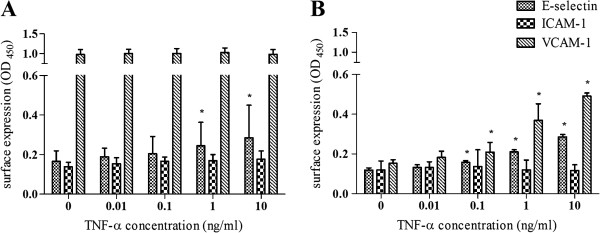
**Surface expression of cell adhesion molecules on EC lines in response to TNF-α.** Immortalized aortic **(A)** or venous **(B)** endothelial cells were incubated for 6 h (E-selectin) or 12 h (ICAM-1 and VCAM-1) with either vehicle control or TNF-α at indicated doses. Surface expression was measured by ELISA as described in materials and methods. OD_450_, absorbance units at 450 nm. Bars show means with SD from 3 separate experiments. * Significantly different from control expression, P < 0.05.

To estimate the immunologic activity of the transformed ECs, the surface expression of E-selectin, ICAM-1 and VCAM-1 in response to TNF-α was examined using a cell-based ELISA (Figure 5). E-selectin was not expressed on unstimulated arterial nor venous transformed ECs, as similar OD-values were obtained when the primary antibody was substituted with an isotype matched irrelevant monoclonal antibody (data not shown). Upon EC activation, E-selectin was induced in a dose-dependent manner on both cell lines. Over a period of 6 h, TNF-α (10 ng/ml) caused 1.7 and 2.4-fold increases in surface expression compared with basal expression on arterial and venous ECs, respectively. We observed a complete loss of both constitutive as inducible expression of ICAM-1 on both cell lines. ELISA analysis of resting immortalized venous ECs demonstrated a negligible basal expression of VCAM-1 and this expression was threefold up regulated after a stimulation period of 12 h with TNF-α (10 ng/ml). In contrast, immortalized aortic ECs showed a high basal expression of VCAM-1 that was almost two times higher than the maximum *de novo* expression on venous ECs. In addition, the VCAM-1 expression on the aortic ECs was not further increased when exposed to TNF-α.

## Discussion

Endothelial cells are active participants in a wide variety of physiological and pathological processes. The ability to isolate and culture ECs has add tremendously to our understanding regarding their importance in these processes. Here, we have described in detail reliable and relatively simple protocols for the isolation and culture of ECs from feline aorta and vena cava. Yet, as described by many others, the use of these primary ECs in cell culture technology is confined by their inherently short replicative life span, their fastidious culture requirements and their lot-to-lot variability. These limitations have prompted us to undertake the immortalization of these primary cells to generate cell lines that provide a consistent model system for the study of the vascular endothelium. In this study, we have established two novel immortalized feline endothelial cell lines that retained much of the phenotypic characteristics of normal ECs and the capacity to recapitulate endothelial functions. To the best of our knowledge, this is the first report on the transformation of feline ECs by sequential lentiviral transduction with SV40LT and hTERT genes. Primary ECs were first successfully transduced with SV40LT allowing cells to proliferate for many population doublings (PDs) beyond the point at which original cells become senescent. The ability of this viral oncogene to extend the cellular lifespan has been attributed to its competence to abrogate the function of the cell cycle regulators p53 and pRb. This permits cells to escape the first mortality checkpoint known as “replicative senescence”. However, cells that bypass senescence in this way, are still subjected to telomeric shortening and eventually succumb at a second mortality checkpoint referred to as “crisis” [12]. Studies have demonstrated that in a number of cell types, crisis can be circumvented by restoring telomerase activity [18,19]. According to the telomere hypothesis, telomeric repeat DNA at the ends of chromosomes shorten with each cell division in the absence of telomerase activity. Once telomeres have shortened below a preset length, cells activate mechanisms that irreversibly arrest the cell cycle leading to cellular senescence. Telomerase, silenced in most somatic cells, is a ribonuclear protein complex that has a catalytic subunit with reverse transcriptase activity (hTERT) which synthesizes and maintains the telomeres [20,21]. Besides, several studies provide evidence for an additional protective function of telomerase by physical capping of the chromosome ends. This proposed “capping” function prevents cells from inducing a senescence checkpoint by protecting telomeres from recognition as damaged DNA even when they remain short [18,22]. Ectopic expression of hTERT restores telomerase activity giving cells a truly unlimited proliferative potential. Although reconstitution of telomerase activity alone is sufficient to confer immortality on many primary cells, in our hands the hTERT-mediated transformation did not render the feline primary ECs immortalized (data not shown). On the other hand, the association of SV40LT and hTERT gene expression was effective to drive these cells continuously through the cell cycle. This reinforces the observation of others that additional changes, such as inactivation of pRb and p53, are a pre-requisite for hTERT-induced immortalization [23,24]. At the time of writing, the feline EC lines have been cultured for more than 30 passages and none of them show signs of growth arrest or cell death. On top, transformation of the EC isolates released the cells from their requirements for pre-formed extracellular matrices, exogenous growth factors and high serum concentrations. These data clearly demonstrate that the feline ECs are immortalized and can serve as continuously renewable cell lines that can be cultured in simple growth medium. Nevertheless, viral oncoproteins have pleiotropic effects on cell physiology and can confer altered geno-and phenotype to the immortalized cells [25]. Therefore, before utilizing the EC lines as substitutes for primary ECs *in vitro*, it remained to be demonstrated that the immortalized cells retained much of the normal physiology of ECs. For this end, phenotypical and functional assays were carried out to directly compare the transformed cells with their parental cells. Since no single endothelial characteristic is sufficient to confirm endothelial identity, the best means of characterizing endothelial cultures is to examine a series of properties and then to make an assessment. Characteristic, although not definitive, endothelial features include morphology, vWF expression, strong uptake of DiI-Ac-LDL and staining with UEA-1 lectins [26]. Feline EC lines were practically similar to the primary cells in terms of morphological appearance, possession of scavenger receptors (DiI-Ac-LDL endocytosis), and presence of α-L-fucosyl containing glycoproteins on the surface (UEA-1 binding). Yet, we failed to detect the presence of vWF antigen in the two continuous EC lines, whereas the original EC cultures were strongly labeled with antiserum against this glycoprotein. Since viral oncoproteins induce chromosomal aberrations in the host cell, it is possible that a highly differentiated function, as the production of vWF, is abolished by the immortalization procedure. However, as reported by Müller *et al.*, the limitation of a monolayer cell culture, deprived of essential physiological factors such as shear stress or the presence of other cell types, must be taken into consideration as an alternative, more likely explanation [27]. Indeed, it is known that vWF synthesis is controlled at the transcriptional level in response to the tissue microenvironment [28]. The observation that the non-transformed ECs lose their ability to express significant levels of this molecule after already the first passage also supports this idea.

To constitute a relevant model for addressing specific issues of endothelial cell biology, the model system must not only closely resemble the phenotype of the original primary cells but also exhibit specific EC functions. Therefore, two typical endothelial functions, capillary-like tube formation on matrigel and the ability to mobilize adhesion molecules in response to a pro-inflammatory stimulus, were used to confirm functionality. ECs are the primary cells involved in the formation of new blood vessels or angiogenesis and this can be mimicked *in vitro* by culturing cells on matrigel [29]. Non-transformed ECs, placed on matrigel, formed branching tube-like structures within 24 h. Despite transformation that has conferred on the EC lines, they also clearly responded to this extracellular matrix message as they were capable of generating vascular-like channels on matrigel within 12 h.

Endothelial cells are key regulators of the inflammatory response by controlling leukocyte recruitment. Cytokines and other inflammatory mediators induce cell adhesion molecules on the surface of ECs which allow leukocytes to recognize sites of inflammation and bind the blood vessel wall [30]. We examined the capacity of the immortalized ECs to upregulate the expression of cell adhesion molecules E-selectin, ICAM-1 and VCAM-1 following their stimulation with a pro-inflammatory stimuli. TNF-α was used because it is a potent stimulator for E-selectin, ICAM-1 and VCAM-1 expression and it is produced during many inflammatory processes [31]. TNF-α could elicit the characteristic up-regulation of E-selectin and VCAM-1 on venous immortalized ECs in a dose-dependent manner. These results demonstrate that the venous EC line retained its typical response to pro-inflammatory stimulation. Aortic immortalized ECs behaved functionally in a manner similar to the venous ECs with respect to the cytokine-inducible expression of E-selectin. However, the aortic EC line exhibited a high constitutive surface expression of VCAM-1 that was not further induced upon activation. These differences may reflect the heterogeneity of ECs arising from different vascular compartments, as high VCAM-1 expression on arterial ECs may be pivotal to leukocyte recruitment in regions subjected to major hemodynamic stress. However, differences may also be imposed by the immortalization procedure. Both cell lines showed a complete loss of both constitutive as inducible ICAM-1 expression. In further attempts to induce ICAM-1 expression, shorter (4 h) and longer (48 h) time periods of TNF-α (10 ng/ml) treatment and interleukine- 1 (Il-1; 10 ng/ml) and lipopolysaccharide (LPS; 1 μg/ml) stimulation were tested. No ICAM-1 expression was noted 4 h or 48 h after TNF-α treatment. Treatments with Il-1 and LPS for 12 h failed to induce ICAM-1 expression on both EC lines (data not shown). The absence of ICAM-1 expression on the continuous EC lines cannot be explained by a TNF-α-receptor defect as this stimuli elicited enhanced expression of E-selectin and VCAM-1. As it was not possible to perform the assay with primary ECs, we cannot exclude that non-transformed feline ECs in culture lack ICAM-1 expression as well. Leukocyte recruitment involves multiple steps, including initial leukocyte rolling along the endothelium, firm adhesion and activation, and ultimately transmigration through the endothelium. Each step is controlled by partially constitutive, partially dynamically regulated complementary adhesion molecules expressed on the surface of both participating cells. Therefore, it would be of great interest to examine the expression of other adhesion molecules that are also involved in leukocyte extravasation, e.g. P-selectin, ICAM-2, and platelet endothelial cell adhesion molecule-1 (PECAM-1).

All these data collectively support that the EC lines retained nearly all phenotypic and functional characteristics of endothelial cells as defined by essentially and commonly accepted criteria.

## Conclusion

In summary, we have described in detail the methodology for the successful isolation and culture of feline endothelial cells from aorta and vena cava. By ectopic expression of SV40LT and hTERT we have generated EC lines from these primary EC cultures. The induced transformation preserved most of the features of vascular endothelium. The ready availability of well-characterized feline EC lines opens the door for detailed *in vitro* studies and will allow the dissection of fundamental mechanisms of endothelial bioactivity and endothelial-related diseases in cats and humans.

## Competing interests

The authors declare that they have no competing interests.

## Authors’ contributions

DAJO participated in the design of the study, carried out cell isolation, characterization and immortalization processes, analyzed and interpreted the results and drafted the manuscript. LMBD participated in cell characterization and immortalization. AD participated in the analysis, interpretation and presentation of data. HLD contributed in the design of the study. HJN designed and supervised the project, contributed in the interpretation of data and critically commented on the manuscript at all stages. All authors have read and approved the final manuscript.
